# Master athletes have higher miR-7, SIRT3 and SOD2 expression in skeletal muscle than age-matched sedentary controls

**DOI:** 10.1016/j.redox.2018.07.022

**Published:** 2018-08-07

**Authors:** Erika Koltai, Zoltan Bori, Peter Osvath, Ferenc Ihasz, Szablics Peter, Geza Toth, Hans Degens, Jörn Rittweger, Istvan Boldogh, Zsolt Radak

**Affiliations:** a Research Institute of Sport Science, University of Physical Education, Budapest, Hungary; b Department of Health Sciences and Sports Medicine, University of Physical Education, Budapest, Hungary; c Hungary Institute of Sport Science, Faculty of Education and Psychology, Eotvos University, Szombathely, Hungary; d Institute of Physical Education and Sport Science, JGYPK, University of Szeged, Szeged, Hungary; e Affidea Diagnostic Center, Budapest, Hungary; f School of Healthcare Science, Manchester Metropolitan University, UK; g Institute of Sport Science and Innovations, Lithuanian Sports University, Lithuania; h Division Space Physiology, Institute of Aerospace Medicine, German Aerospace Center, Cologne, Germany; i University of Texas Medical Branch at Galveston, Galveston, TX 77555, USA

**Keywords:** Master athlete, miRNA, Skeletal muscle, Anti-oxidant

## Abstract

Regular physical exercise has health benefits and can prevent some of the ageing-associated muscle deteriorations. However, the biochemical mechanisms underlying this exercise benefit, especially in human tissues, are not well known. To investigate, we assessed this using miRNA profiling, mRNA and protein levels of anti-oxidant and metabolic proteins in the vastus lateralis muscle of master athletes aged over 65 years and age-matched controls. Master athletes had lower levels of miR-7, while mRNA or protein levels of SIRT3, SIRT1, SOD2, and FOXO1 levels were significantly higher in the vastus lateralis muscle of master athletes compared to muscles of age-matched controls. These results suggest that regular exercise results in better cellular metabolism and antioxidant capacity via maintaining physiological state of mitochondria and efficient ATP production and decreasing ageing-related inflammation as indicated by the lower level of miR-7 in master athletes.

## 1 Introduction

Skeletal muscle is the most abundant tissue in human body, accounting for about 60% of the total protein content and 40% of body mass. It is not only important for locomotion and maintenance of body posture, but also has an important metabolic function such as storage of carbohydrates in the form of glycogen. Aging results in significant loss in muscle mass and function, which directly effects well-being and mortality [1]. The loss of muscle mass is attributable to a gradual decline in the number of muscle fibers that begins around the age of 50, where by the age of 80 approximately 50% of the fibers are lost [2]. Physical exercise might be the only natural tool to attenuate sarcopenia. Indeed, regular exercise has been shown to be associated with larger muscle cross sectional area [3], fiber number [4], [5], strength [6], endurance capacity [7], mitochondrial function [8], insulin sensitivity [9], among others.

Aging is associated with alterations in the miRNA profile in skeletal muscle [10], [11] and deterioration of mitochondrial function dynamics [12], [13], [14]. Physical activity induces a wide range of functional and biochemical changes in skeletal muscle including epigenetic changes, such as alterations in the miRNA profile [15], [16], [17], [18]. For instance, both short- [19] and long-term endurance exercise [20] induced changes in the levels of a number of miRNAs that are involved in the regulation of skeletal muscle regeneration, gene expression and mitochondrial biogenesis. These effects of endurance training are not limited to healthy people, but also in patients with polymyositis or dermatomyositis endurance exercise induced an increase in muscle miRNA levels that target transcripts involved in inflammation, metabolism and muscle atrophy [21]. Hypertrophic stimuli can also induce changes in miRNA levels as illustrated by our observation that functional mechanical overloading by synergist muscle ablation induced alterations in miRNA levels that control atrophy and hypertrophy [22]. It is thus possible that regular exercise can reverse some of the detrimental ageing-related changes in the miRNA profile and mitochondrial function.

Since ageing is associated with reduced levels of physical activity [23] and disuse does cause muscle wasting and reductions in oxidative capacity [24] master athletes (athletes 35 years and older) may provide an excellent model to study the effects of ageing per se on muscle, not confounded by disuse [25]. Therefore, the purpose of this investigation was to study the effects differences in the miRNA and mitochondrial profile between master athletes aged over 65 years old and age-matched controls.

## 2 Methods

### 2.1 Participants

We recruited 26 Master Athletes (10 female and 16 male) at the European Veterans Athletics Championships in 2010 (Nyíregyháza, Hungary). Control participants (n = 18, 13 female, 5 male) were also recruited. The participants provided written informed consent before inclusion. For this study we have selected 10 master athletes 65 ± 5 years and 13 sedentary subjects 64.67 ± 2.08 years old. The master athletes reported that they all had been training for more than sixty years, while control subjects were sedentary.

The investigation was approved by the local ethics committee (File number: 10826-0/2010-1018EKU, research permission number: 15/07/2010-24/07/2010) and performed in compliance with the Declaration of Helsinki.

### 2.2 Muscle biopsy

Muscle biopsies were obtained from the vastus lateralis using a conchotome or needle biopsy technique as described earlier [26]. Samples were frozen in liquid nitrogen and stored at − 80 °C until biochemical analysis.

### 2.3 RNA isolation

Total RNA, including miRNA, was isolated from muscle biopsy samples by miRNeasy Mini Kit (Qiagen #217004) according to the instructions of the manufacturer.

### 2.4 miRNA microarray analysis

miRNA expression analysis was performed on 4 samples from master athletes (68.75 ± 8.54 years old) and 4 samples of sedentary subjects (70.25 ± 11.3 years old) gained by skeletal muscle biopsy samples with Agilent Human miRNA Microarray Release 14.0 8 × 15K resolution array (Agilent Technologies, USA), that distinguishes 887 human miRNAs. The microarray was performed according to the instructions by the manufacturer (Agilent miRNA microarray protocol 2.4). Hundred ng of total RNA were dephosphorylated and marked with Cyanine-3-pCp dye using the miRNA Complete Labeling and Hyb Kit (Agilent Technologies, USA). Purification of the marked RNA was performed by Micro Bio-Spin P-6 column (Bio-Rad Laboratories; Hercules, CA, USA) and then hybridized onto the Human miRNA Microarray Release 14.0 microarray slides. After hybridization, slides were washed at room temperature and scanned using an Agilent DNA microarray scanner. Raw data were extracted with the Agilent Feature Extraction Software 11.0.

### 2.5 Detection of mature microRNAs in skeletal muscle

The TaqMan miRNA reverse transcriptase kit and TaqMan miRNA assays (Applied Biosystems, Foster City, CA) were used to quantify mature miRNA expression levels. Each target miRNA was quantified according to the manufacturer's protocol with minor modifications. Briefly, reverse transcriptase reactions were performed with miRNA-specific reverse transcriptase primers and 5 ng of purified total RNA for 30 min at 16 °C, 30 min at 42 °C, and finally 5 min at 85 °C to heat-inactivate the reverse transcriptase. All volumes suggested in the manufacturer's protocol were halved, as previously reported [27]. Real-time PCRs for each miRNA (10 μl total volumes) were performed in triplicate, and each 10-μl reaction mixture included 2.4 μl of 10×-diluted reverse transcriptase product. Reactions were run on a PRISM 7900HT Fast Real-Time PCR System (Applied Biosystems) at 95 °C for 10 min, followed by 40 cycles at 95 °C for 15 s and 60 °C for 1 min. Twofold dilution series were performed for all target miRNAs to verify the linearity of the assay. To account for possible differences in the amount of starting RNA, all samples were normalized to miR-423. All reactions were run singleplex and quantified using the cycle threshold (ΔΔC_t_) method [28].

### 2.6 mRNA expression levels

#### 2.6.1 cDNA synthesis

cDNA was synthesized using a Tetro cDNA Synthesis kit (Bioline #BIO-65026 Luckenwalde, Germany) in accordance with the manufactures’ instructions. Briefly, the reaction conditions were as follows: 1 μg of RNA, 1 μl of random primers, 1 μl of 10 mM dNTP, 1 μl of RNase inhibitor, and 0.25 μl of 200 U/μl reverse transcriptase in a final volume of 20 μl. The solution was incubated for 10 min at 25 °C for primer annealing, followed by 42 °C for 60 min for primer elongation, and followed by 80 °C for 5 min termination. cDNA samples were stored at − 20 °C.

##### 2.6.1.1 Real time quantitative RT-PCR (qRT-PCR) reaction

Based on the principle of the SybrGreen detection method, EvaGreen® dye (Biotium, Hayward, CA, USA) was used to detect PCR products. The PCR was performed using a primer pair specific for mRNA of vascular endothelial growth factor (VEGF), silent mating type information regulation 2 homolog 1 (SIRT1), forkhead box protein O1 (FOXO1), mitochondrial calcium uniporter (MCU, Insulin like growth factor (IGF-1), peroxisome proliferator-activated receptor gamma coactivator 1-alpha (PGC1α) and mechano growth factors (MGF) isoforms (for sequences of mRNA genes used in the study see Table 1). PCR amplifications consisted of equal amounts of template DNA, 10 μl of ImmoMix™ complete ready-to-use heat-activated 2× reaction mix (Bioline GmbH, Luckenwalde, Germany), 1 μl of 20x EvaGreen (Biotium, Hayward, CA, USA), 2.5 μl of 10 nmol/L forward and reverse primer (IBAGmbH, Göttingen, Germany) and water to a final volume of 20 μl. Amplifications were performed in a Rotor-Gene 6000 thermal cycler (Corbett Life Science/Qiagen, London, UK) at 95 °C for 10 min, followed by 40 cycles of 95 °C for 10 s, 60 °C for 20 s and 72 °C for 30 s in triplicates. The validity of the signal was evaluated by melting analysis and agarose gel electrophoresis. Human 28 S rRNA gene served as an endogenous control gene (Table 1).

**Table 1:** Reference genes.

**H-28S-F**	AGCCGATCCATCATCCGCAATG
**H-28S-R**	CAGCCAAGCTCAGCGCAAC
**H-FOXO1-F**	AAGAGCGTGCCCTACTTCAA
**H-FOXO1-R**	CATCCCCTTCTCCAAGATCA
**H-IGF1/MGF-F**	CGAAGTCTCAGAGAAGGAAAGG
**H-IGF1/MGF-R**	ACAGGTAACTCGTGCAGAGC
**H-IGF1-F**	GCTCTTCAGTTCGTGTGTGGA
**H-IGF1-R**	GCCTCCTTAGATCACAGCTCC
**H-MCU-F**	CACTGTTGTGCCCTCTGATG
**H-MCU-R**	ACTCTGTCAATTCCCCGATCC
**H-PPARGC1A (PGC-1a)-F**	GTGAAGACCAGCCTCTTTGC
**H-PPARGC1A (PGC-1a)-R**	TCACGTCTCCATCTGTCAGC
**H-SIRT1-F**	TGCGGGAATCCAAAGGATAATTCAGTGTC
**H-SIRT1-R**	CTTCATCTTTGTCATACTTCATGGCTCTATG
**H-VEGFA-F**	AGGAGGAGGGCAGAATCATCA
**H-VEGFA-R**	CTCGATTGGATGGCAGTAGCT

### 2.7 Western blots

Tissue homogenates of the muscle biopsy samples were generated with an Ultra Turrax® (IKA®-Werke) homogenizer using 10 vol of lysis buffer (137 mM NaCl, 20 mM Tris-HCl, pH 8.0, 2% NP-40, 10% glycerol and protease inhibitors). Five to ten micrograms of protein were electrophoresed on 10–12% v/v polyacrylamide SDS-PAGE gels. Proteins were electrotransferred onto polyvinylidene difluoride (PVDF) membranes. The membranes were subsequently blocked in 0.5% BSA, and after blocking incubated with primary antibodies (SIRT3 1:2500 Abcam #ab40006, SOD2 1:2500 Sigma-Aldrich #SAB1406465, Cytochrome oxidase4 (COX4) (D-20) 1:2500 Santa Cruz #sc-69359, GAPDH 1:50000 Sigma-Aldrich #G8795) overnight at 4 °C. After incubation with primary antibodies, membranes were washed in tris-buffered saline-Tween-20 (TBST) and incubated with HRP-conjugated secondary antibodies (1:50000, Jackson ImmunoResearch Europe Ltd). After incubation with secondary antibodies, membranes were repeatedly washed. Membranes were incubated with chemiluminescent substrate (Thermo Scientific, SuperSignal West Pico Chemiluminescent Substrate #34080) and protein bands were visualized on X-ray films. The bands were quantified by ImageJ software, and normalized to GAPDH, which served as an internal control.

### 2.8 Statistical analyses

Data gathered from the miRNA array validation and gene expression experiments were analyzed with an unpaired Mann-Whitney *U*-test, and unpaired, two-tailed Student's *t*-test or *χ*^2^ test were used for qPCR and Western blot variables, as appropriate. Data are presented as mean ± standard deviation. Significance level was set at *p* < 0.05.

## 3 Results

**Fig. 1:**
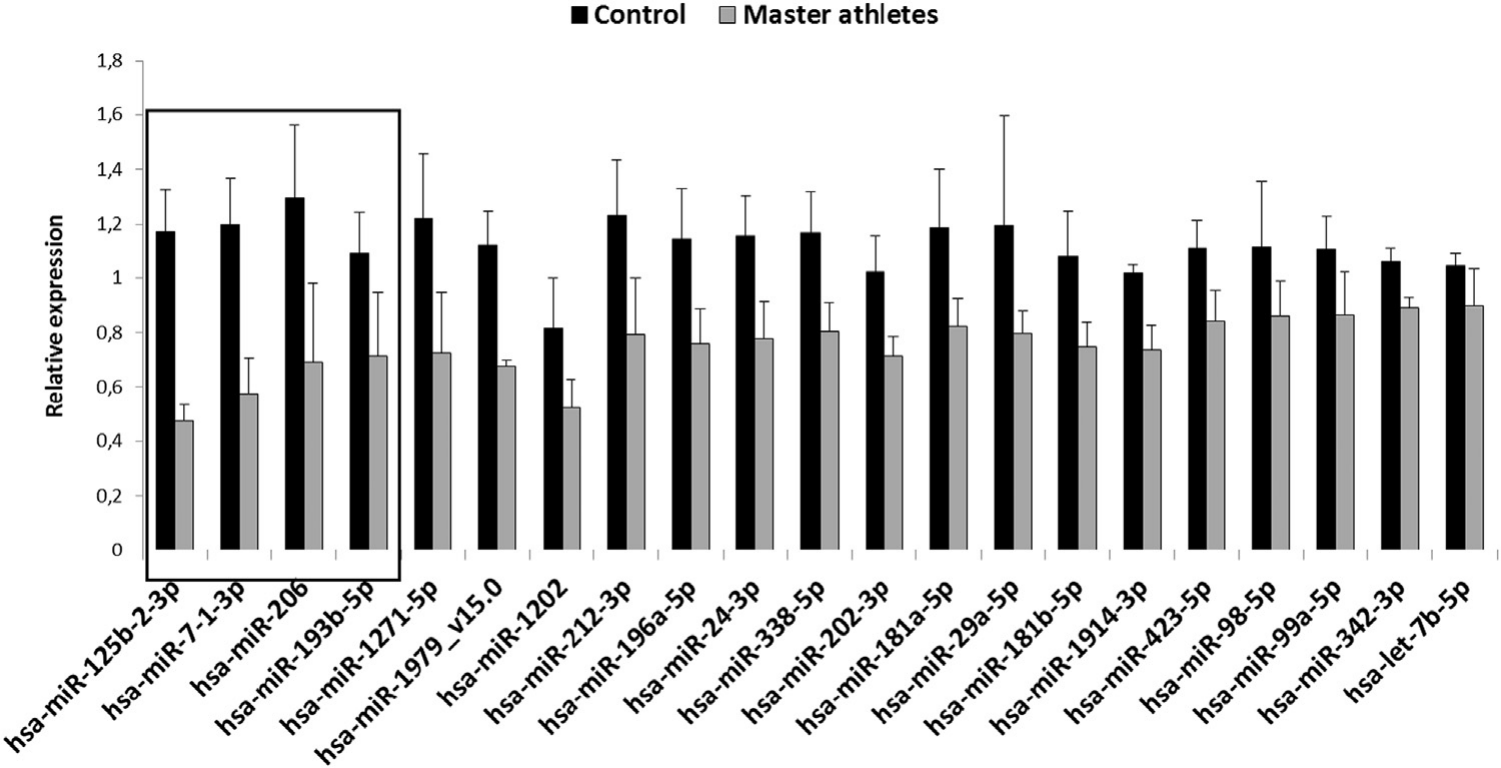
miRNA array profile of master athletes and sedentary subjects, The array screened for 887 miRNA and 21 of them showed significant difference (p < 0.05) between sedentary and master athletes. Results are expressed mean ± SD, N = 4 in each group.

**Fig. 2:**
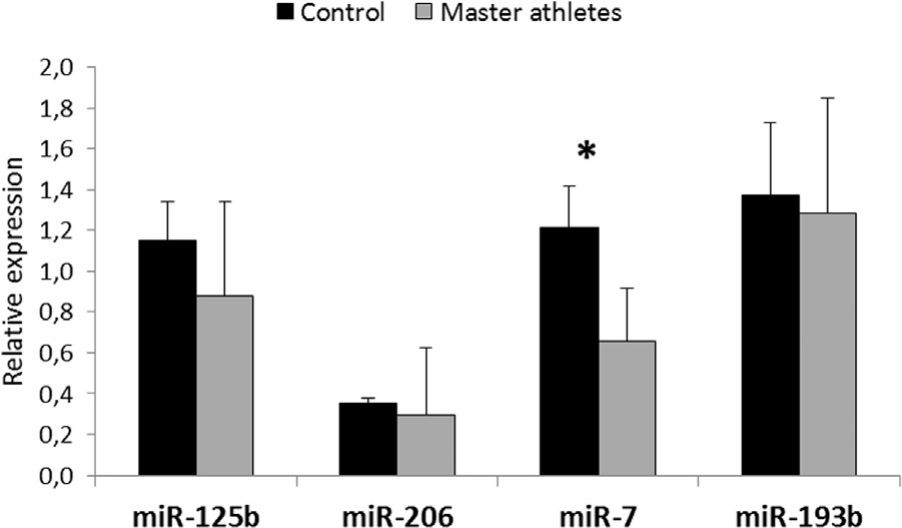
q-PCR results of miRNA levels, Four microRNA were selected to q-PCR measurements and only miR-7 analysis showed significant difference. Results are expressed mean ± SD, N = 4 in each group, *p < 0.05.

First we performed miRNA array from the biopsy muscle samples of Master athletes and control subjects. The microarray analysis revealed that 21 of the 887 miRNA sequences were lower in the master athlete than control muscles (Fig. 1). Four miRNA were selected based on the greatest difference in the miRNA array (indicated in the box in Fig. 1) for further q-PCR analysis. This revealed that only miR-7 was expressed more (p < 0.05) in the muscles from controls than in those from master athletes (Fig. 2).

**Fig. 3:**
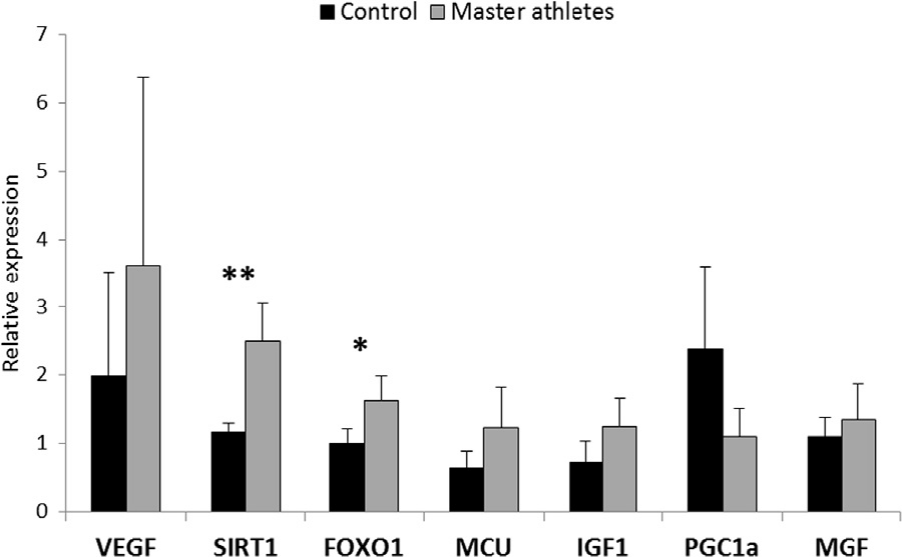
mRNA levels of selected regulatory proteins in master athletes and sedentary subjects. The mRNA levels of seven key proteins were studied and SIRT1 and FOXO1 mRNA levels were significantly higher in skeletal muscle of master athletes than is sedentary subjects. Results are expressed mean ± SD, N = 10 in master athletes and N = 13 in controls group. ** p < 0.01, *p < 0.05.

**Fig. 4:**
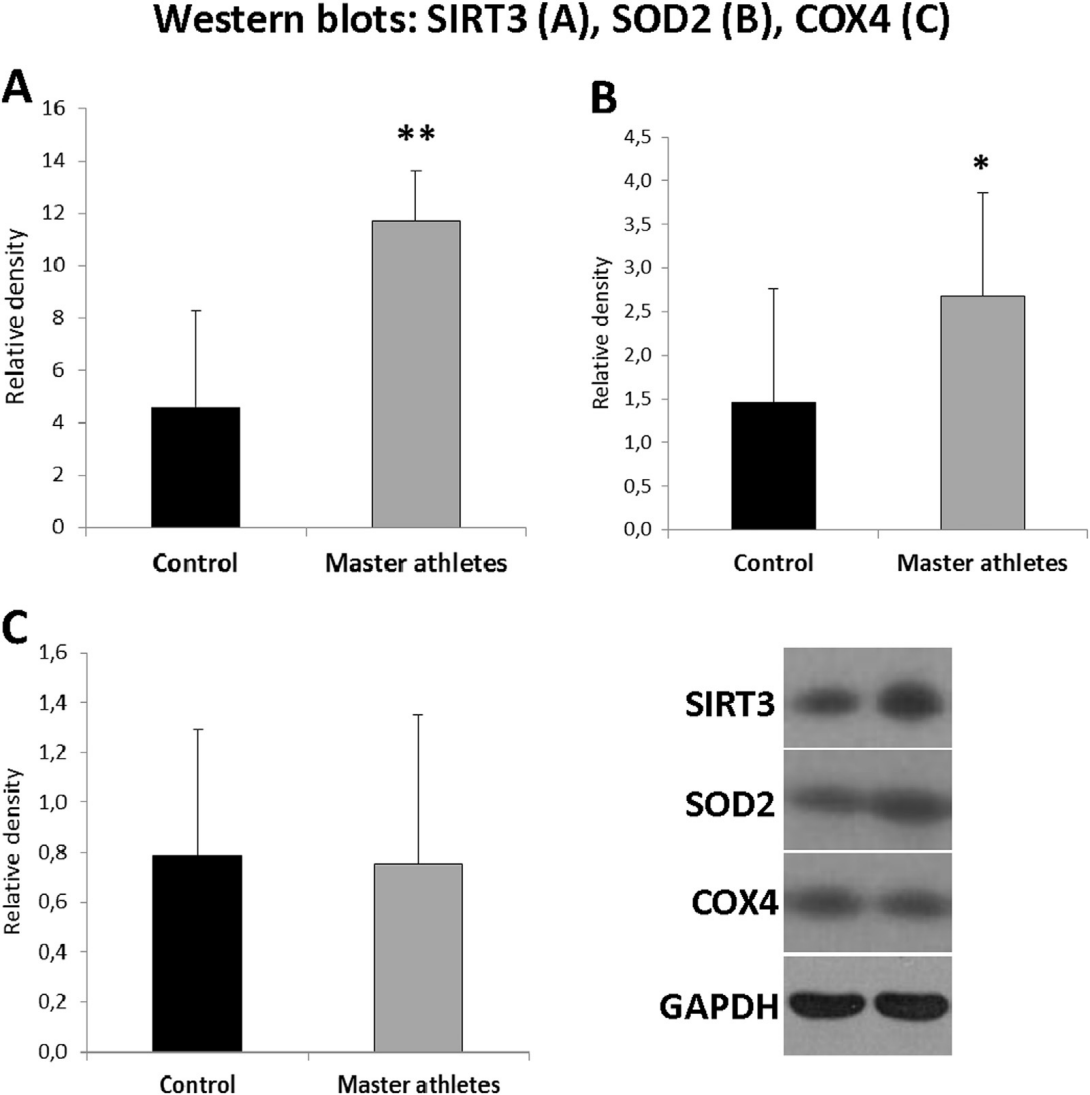
Protein levels of SIRT3, SOD2 and COX4, immunoblot data revealed that SIRT3 and SOD2 levels of master athletes were significantly elevated compared to controls. Results are expressed mean ± SD, N = 10 in master athletes and N = 13 in controls group. ** p < 0.01, *p < 0.05.

Then from the remained muscle samples, key mitochondrial mRNA (Fig. 3) and protein (Fig. 4) contents were measured. SIRT1 (p < 0.01) and FOXO1 (p < 0.05) mRNA levels were higher in master athletes than in control groups (Fig. 3), while the SIRT3 and SOD2 proteins (p < 0.01; Fig. 4) from the muscle samples of master athletes were higher than that in the control subjects.

## 4 Discussion

Aging is associated with an increased level of miR-7 and has been shown to play a crucial role in ageing-associated loss of transforming growth factor-beta 1 dependent fibroblast to myofibroblast differentiation, and hence poorer wound healing [29]. It was proposed that miR-7 up-regulation in aged cells reduced the expression of epidermal growth factor receptor (EGFR) protein via degradation of its mRNA, but it may also interact with the mRNA of downstream targets of the EGFR-dependent signaling pathway such as MAPK/ERK, CaMKII, Rho-GTPase, PI3K, Akt and mTOR [29]. This signaling pathway is important to wound healing in skeletal muscle [30]. The significance of miR-7 for fibroblast function is also illustrated by the diminished miR-7 level after estradiol treatment that resulted increased EGFR mRNA expression and restored functionality of aging fibroblasts [31]. The cause of the senescent state of the fibroblasts and elevated miR-7 levels in old age has been suggested to be involved in chronic inflammation and specifically the interferon-linked pathway [31]. In line with the role of systemic inflammation to induce miR-7 is the observation that miR-7 expression is also elevated in airways of patients suffering from allergic rhinitis [32] and chronic obstructive pulmonary disease [33], and in peripheral blood mononuclear cells of HIV patient [34], conditions associated with local or systemic inflammation. Moreover, it is known that facioscapulohumeral muscular dystrophy is also associated with both inflammation [35] and elevated miR-7 expression in muscle [36]. It is thus possible that sarcopenia-associated inflammation may at least partly contribute to the expression of miR-7 in old muscle. Exercise may reduce systemic inflammation and expression of inflammatory markers in muscle [37], the anti-inflammatory effect of life-long exercise may thus explain the lower miR-7 levels, we observed in the muscles of our master athletes than in those of non-athletes.

Besides the role of miR-7 in inflammation, it seems that it is also important to lipid metabolism. It has been shown that miR-7 mediates cross-talk between peroxisome proliferator-activated receptor (PPAR), sterol regulatory element-binding proteins (SREBP), and liver X receptors signaling pathways [38]. PPAR-α signaling regulates miR-7, which activates SREBP. Down-regulation of miR-7 is associated with sebaceous lipogenesis [39]. It is known that high level endurance performance needs a high level of energy supply. Indeed, in mouse model, which was developed to study extreme endurance, mice have significantly elevated levels of PPAR-α and lipogenesis [40], suggesting, the regular exercise mediated metabolic challenge could involve miR-7-mediated up-regulation of fat metabolism.

Another main finding of this study was, that in muscles from people who performed life-long exercise SIRT3 protein levels were higher than in the skeletal muscle of sedentary people. SIRT3 has a powerful regulatory role in lipid metabolism. SIRT3 ablation exhibits hallmarks of fatty-acid oxidation disorders during fasting, including reduced ATP levels [41]. Indeed, it has been shown that SIRT3 controls fatty acid metabolism by deacetylation of medium-chain acyl-CoA dehydrogenase and acyl-CoA dehydrogenase [42], therefore ablation of SIRT3 would highly impact lipid metabolism. Moreover, it has been shown that SIRT3 can interact and deacetylate ATP-synthase F-complex [43], hence SIRT3 directly controls ATP production. This explains why SIRT3 knock-out mice have decreased production of ATP. In addition, it is also known that the aging is associated with decline in SIRT3 levels [44], which can explain the age related decline in ATP production. Here we show that life-long physical exercise significantly increases SIRT3 levels and this is a powerful beneficial effect of exercise against age-associated functional deterioration of mitochondria. SIRT3 deacetylates two critical lysine residues on SOD2 and promotes its antioxidant activity, and decreases the level of ROS in the mitochondria [45]. Therefore, physical exercise through increase in SIRT3 level and activation of SOD2 can attenuate the age-associated decline in mitochondrial function and suppress oxidative stress [46].

Due to the limited amount of samples, we could select just some important proteins in the skeletal muscle to investigate the effects of life-long exercise training. According to our results the mRNA levels of SIRT1 and FOXO1 were elevated in the muscle of master athletes compared to control subjects. We and others have shown that exercise training can prevent the age-related decrease in the level and activity of SIRT1 and the associated functional alterations [12], [17], [47], [48]. FOXO1 is involved in glycolytic and lipolytic flux, and mitochondrial metabolism, thus it is important part of adaptive response to cope with energy challenge during exercise [49]. Moreover, it was suggested that the deacetylation of FOXO1 by SIRT3 elevates the expression of the FOXO1 target genes, like SOD2 while decreasing senescence phenotypes [50].

In conclusion, our data suggest that life-long exercise program results in down-regulation of miR-7 in skeletal muscle of master athletes, which can lead to suppression of sarcopenia related inflammation, better fat metabolism. The increased level of SIRT3 supports more efficient fat metabolism, ATP production and antioxidant capacity through SOD2 in skeletal muscle of master athletes compared to controls subjects. Life-long exercise attenuates the age-associated decline in energy metabolism and antioxidant systems in the skeletal muscle.

## References

[1] McLeod M., Breen L., Hamilton D.L., Philp A. (2016). Live strong and prosper: the importance of skeletal muscle strength for healthy ageing. Biogerontology.

[2] Faulkner J.A., Larkin L.M., Claflin D.R., Brooks S.V. (2007). Age-related changes in the structure and function of skeletal muscles. Clin. Exp. Pharmacol. Physiol..

[3] Narici M.V., Reeves N.D., Morse C.I., Maganaris C.N. (2004). Muscular adaptations to resistance exercise in the elderly. J. Musculoskelet. Neuron. Interact..

[4] Power G.A., Allen M.D., Gilmore K.J., Stashuk D.W., Doherty T.J., Hepple R.T., Taivassalo T., Rice C.L. (1985). Motor unit number and transmission stability in octogenarian world class athletes: can age-related deficits be outrun?. J. Appl. Physiol..

[5] Aagaard P., Suetta C., Caserotti P., Magnusson S.P., Kjaer M. (2010). Role of the nervous system in sarcopenia and muscle atrophy with aging: strength training as a countermeasure. Scand. J. Med. Sci. Sports.

[6] Englund D.A., Kirn D.R., Koochek A., Zhu H., Travison T.G., Reid K.F., von Berens A., Melin M., Cederholm T., Gustafsson T., Fielding R.A. (2017). Nutritional supplementation with physical activity improves muscle composition in mobility-limited older adults, the VIVE2 study: a randomized, double-blind, placebo-controlled trial. J. Gerontol. A Biol. Sci. Med. Sci..

[7] Radak Z., Naito H., Kaneko T., Tahara S., Nakamoto H., Takahashi R., Cardozo-Pelaez F., Goto S. (2002). Exercise training decreases DNA damage and increases DNA repair and resistance against oxidative stress of proteins in aged rat skeletal muscle. Pflug. Arch..

[8] Hood D.A., Tryon L.D., Carter H.N., Kim Y., Chen C.C. (2016). Unravelling the mechanisms regulating muscle mitochondrial biogenesis. Biochem. J..

[9] Sogaard D., Lund M.T., Scheuer C.M., Dehlbaek M.S., Dideriksen S.G., Abildskov C.V., Christensen K.K., Dohlmann T.L., Larsen S., Vigelso A.H., Dela F., Helge J.W. (2017). High-intensity interval training improves insulin sensitivity in older individuals. Acta Physiol..

[10] Raz V., Riaz M., Tatum Z., Kielbasa S.M., Hoen P.A.C. (2017). The distinct transcriptomes of slow and fast adult muscles are delineated by noncoding RNAs. FASEB J..

[11] McCormick R., Goljanek-Whysall K. (2017). MicroRNA dysregulation in aging and pathologies of the skeletal muscle. Int. Rev. Cell Mol. Biol..

[12] Koltai E., Hart N., Taylor A.W., Goto S., Ngo J.K., Davies K.J., Radak Z. (2012). Age-associated declines in mitochondrial biogenesis and protein quality control factors are minimized by exercise training. Am. J. Physiol. Regul. Integr. Comp. Physiol..

[13] Drake J.C., Yan Z. (2017). Mitophagy in maintaining skeletal muscle mitochondrial proteostasis and metabolic health with ageing. J. Physiol..

[14] Kim Y., Triolo M., Hood D.A. (2017). Impact of aging and exercise on mitochondrial quality control in skeletal muscle. Oxid. Med. Cell. Longev..

[15] Ntanasis-Stathopoulos J., Tzanninis J.G., Philippou A., Koutsilieris M. (2013). Epigenetic regulation on gene expression induced by physical exercise. J. Musculoskelet. Neuron. Interact..

[16] Silva G.J.J., Bye A., El Azzouzi H., Wisloff U. (2017). MicroRNAs as important regulators of exercise adaptation. Prog. Cardiovasc. Dis..

[17] Koltai E., Szabo Z., Atalay M., Boldogh I., Naito H., Goto S., Nyakas C., Radak Z. (2010). Exercise alters SIRT1, SIRT6, NAD and NAMPT levels in skeletal muscle of aged rats. Mech. Ageing Dev..

[18] Moreira O.C., Estebanez B., Martinez-Florez S., de Paz J.A., Cuevas M.J., Gonzalez-Gallego J. (2017). Mitochondrial function and mitophagy in the elderly: effects of exercise. Oxid. Med. Cell. Longev..

[19] Russell A.P., Lamon S., Boon H., Wada S., Guller I., Brown E.L., Chibalin A.V., Zierath J.R., Snow R.J., Stepto N., Wadley G.D., Akimoto T. (2013). Regulation of miRNAs in human skeletal muscle following acute endurance exercise and short-term endurance training. J. Physiol..

[20] Nielsen S., Scheele C., Yfanti C., Akerstrom T., Nielsen A.R., Pedersen B.K., Laye M.J. (2010). Muscle specific microRNAs are regulated by endurance exercise in human skeletal muscle. J. Physiol..

[21] Boehler J.F., Hogarth M.W., Barberio M.D., Novak J.S., Ghimbovschi S., Brown K.J., Alemo Munters L., Loell I., Chen Y.W., Gordish-Dressman H., Alexanderson H., Lundberg I.E., Nagaraju K. (2017). Effect of endurance exercise on microRNAs in myositis skeletal muscle-A randomized controlled study. PLoS One.

[22] Koltai E., Bori Z., Chabert C., Dubouchaud H., Naito H., Machida S., Davies K.J., Murlasits Z., Fry A.C., Boldogh I., Radak Z. (2017). SIRT1 may play a crucial role in overload-induced hypertrophy of skeletal muscle. J. Physiol..

[23] Ingram D.K. (2000). Age-related decline in physical activity: generalization to nonhumans. Med. Sci. Sports Exerc..

[24] Degens H., Alway S.E. (2006). Control of muscle size during disuse, disease, and aging. Int. J. Sports Med..

[25] Rittweger J., Kwiet A., Felsenberg D. (2004). Physical performance in aging elite athletes–challenging the limits of physiology. J. Musculoskelet. Neuron. Interact..

[26] Radak Z., Pucsok J., Mecseki S., Csont T., Ferdinandy P. (1999). Muscle soreness-induced reduction in force generation is accompanied by increased nitric oxide content and DNA damage in human skeletal muscle. Free Radic. Biol. Med..

[27] Gallagher I.J., Scheele C., Keller P., Nielsen A.R., Remenyi J., Fischer C.P., Roder K., Babraj J., Wahlestedt C., Hutvagner G., Pedersen B.K., Timmons J.A. (2010). Integration of microRNA changes in vivo identifies novel molecular features of muscle insulin resistance in type 2 diabetes. Genome Med..

[28] Livak K.J., Schmittgen T.D. (2001). Analysis of relative gene expression data using real-time quantitative PCR and the 2(-Delta Delta C(T)) method. Methods.

[29] Midgley A.C., Bowen T., Phillips A.O., Steadman R. (2014). MicroRNA-7 inhibition rescues age-associated loss of epidermal growth factor receptor and hyaluronan-dependent differentiation in fibroblasts. Aging Cell.

[30] Li H.Y., Zhang Q.G., Chen J.W., Chen S.Q., Chen S.Y. (2013). The fibrotic role of phosphatidylinositol-3-kinase/Akt pathway in injured skeletal muscle after acute contusion. Int. J. Sports Med..

[31] Midgley A.C., Morris G., Phillips A.O., Steadman R. (2016). 17beta-estradiol ameliorates age-associated loss of fibroblast function by attenuating IFN-gamma/STAT1-dependent miR-7 upregulation. Aging Cell.

[32] Shaoqing Y., Ruxin Z., Guojun L., Zhiqiang Y., Hua H., Shudong Y., Jie Z. (2011). Microarray analysis of differentially expressed microRNAs in allergic rhinitis. Am. J. Rhinol. Allergy.

[33] Akbas F., Coskunpinar E., Aynaci E., Oltulu Y.M., Yildiz P. (2012). Analysis of serum micro-RNAs as potential biomarker in chronic obstructive pulmonary disease. Exp. Lung Res..

[34] Ballegaard V., Ralfkiaer U., Pedersen K.K., Hove M., Koplev S., Braendstrup P., Ryder L.P., Madsen H.O., Gerstoft J., Gronbaek K., Nielsen S.D. (2017). MicroRNA-210, MicroRNA-331, and MicroRNA-7 are differentially regulated in treated HIV-1-infected individuals and are associated with markers of systemic inflammation. J. Acquir. Immune Defic. Syndr..

[35] Wang L.H., Tawil R. (2016). Facioscapulohumeral dystrophy. Curr. Neurol. Neurosci. Rep..

[36] Dmitriev P., Stankevicins L., Ansseau E., Petrov A., Barat A., Dessen P., Robert T., Turki A., Lazar V., Labourer E., Belayew A., Carnac G., Laoudj-Chenivesse D., Lipinski M., Vassetzky Y.S. (2013). Defective regulation of microRNA target genes in myoblasts from facioscapulohumeral dystrophy patients. J. Biol. Chem..

[37] Degens H. (2010). The role of systemic inflammation in age-related muscle weakness and wasting. Scand. J. Med. Sci. Sports.

[38] Singaravelu R., Quan C., Powdrill M.H., Shaw T.A., Srinivasan P., Lyn R.K., Alonzi R.C., Jones D.M., Filip R., Russell R.S., Pezacki J.P. (2018). MicroRNA-7 mediates cross-talk between metabolic signaling pathways in the liver. Sci. Rep..

[39] Schneider M.R., Samborski A., Bauersachs S., Zouboulis C.C. (2013). Differentially regulated microRNAs during human sebaceous lipogenesis. J. Dermatol. Sci..

[40] Brenmoehl J., Walz C., Renne U., Ponsuksili S., Wolf C., Langhammer M., Schwerin M., Hoeflich A. (2013). Metabolic adaptations in the liver of born long-distance running mice. Med. Sci. Sports Exerc..

[41] Hirschey M.D., Shimazu T., Goetzman E., Jing E., Schwer B., Lombard D.B., Grueter C.A., Harris C., Biddinger S., Ilkayeva O.R., Stevens R.D., Li Y., Saha A.K., Ruderman N.B., Bain J.R., Newgard C.B., Farese R.V., Alt F.W., Kahn C.R., Verdin E. (2010). SIRT3 regulates mitochondrial fatty-acid oxidation by reversible enzyme deacetylation. Nature.

[42] Bharathi S.S., Zhang Y., Mohsen A.W., Uppala R., Balasubramani M., Schreiber E., Uechi G., Beck M.E., Rardin M.J., Vockley J., Verdin E., Gibson B.W., Hirschey M.D., Goetzman E.S. (2013). Sirtuin 3 (SIRT3) protein regulates long-chain acyl-CoA dehydrogenase by deacetylating conserved lysines near the active site. J. Biol. Chem..

[43] Vassilopoulos A., Pennington J.D., Andresson T., Rees D.M., Bosley A.D., Fearnley I.M., Ham A., Flynn C.R., Hill S., Rose K.L., Kim H.S., Deng C.X., Walker J.E., Gius D. (2014). SIRT3 deacetylates ATP synthase F1 complex proteins in response to nutrient- and exercise-induced stress. Antioxid. Redox Signal..

[44] Joseph A.M., Adhihetty P.J., Buford T.W., Wohlgemuth S.E., Lees H.A., Nguyen L.M., Aranda J.M., Sandesara B.D., Pahor M., Manini T.M., Marzetti E., Leeuwenburgh C. (2012). The impact of aging on mitochondrial function and biogenesis pathways in skeletal muscle of sedentary high- and low-functioning elderly individuals. Aging Cell.

[45] Qiu X., Brown K., Hirschey M.D., Verdin E., Chen D. (2010). Calorie restriction reduces oxidative stress by SIRT3-mediated SOD2 activation. Cell Metab..

[46] Joseph A.M., Adhihetty P.J., Leeuwenburgh C. (2016). Beneficial effects of exercise on age-related mitochondrial dysfunction and oxidative stress in skeletal muscle. J. Physiol..

[47] Radak Z., Bori Z., Koltai E., Fatouros I.G., Jamurtas A.Z., Douroudos, Terzis G., Nikolaidis M.G., Chatzinikolaou A., Sovatzidis A., Kumagai S., Naito H., Boldogh I. (2011). Age-dependent changes in 8-oxoguanine-DNA glycosylase activity are modulated by adaptive responses to physical exercise in human skeletal muscle. Free Radic. Biol. Med..

[48] Radak Z., Koltai E., Taylor A.W., Higuchi M., Kumagai S., Ohno H., Goto S., Boldogh I. (2013). Redox-regulating sirtuins in aging, caloric restriction, and exercise. Free Radic. Biol. Med..

[49] Sanchez A.M., Candau R.B., Bernardi H. (2014). FoxO transcription factors: their roles in the maintenance of skeletal muscle homeostasis. Cell Mol. Life Sci..

[50] Zhang B., Cui S., Bai X., Zhuo L., Sun X., Hong Q., Fu B., Wang J., Chen X., Cai G. (2013). SIRT3 overexpression antagonizes high glucose accelerated cellular senescence in human diploid fibroblasts via the SIRT3-FOXO1 signaling pathway. Age.

